# An Analysis of Canadian Institute for Health Research Funding for Research on Autism Spectrum Disorder

**DOI:** 10.1155/2016/8106595

**Published:** 2016-02-10

**Authors:** R. Deonandan, E. Y. Liu, B. Kolisnyk, A. T. M. Konkle

**Affiliations:** ^1^Interdisciplinary School of Health Sciences, Faculty of Health Sciences, University of Ottawa, 25 University Private, THN 208, Ottawa, ON, Canada K1N 6N5; ^2^Department of Public Health Sciences, Queen's University, Kingston, ON, Canada K7L 3N6; ^3^Neuroscience, Schulich School of Medicine and Dentistry, University of Western Ontario, London, ON, Canada N6A 5B7; ^4^School of Psychology, Faculty of Social Sciences, University of Ottawa, Ottawa, ON, Canada K1N 6N5

## Abstract

We examined patterns of Canadian Institute for Health Research (CIHR) funding on autism spectrum disorder (ASD) research. From 1999 to 2013, CIHR funded 190 ASD grants worth $48 million. Biomedical research received 43% of grants (46% of dollars), clinical research 27% (41%), health services 10% (7%), and population health research 8% (3%). The greatest number of grants was given in 2009, but 2003 saw the greatest amount. Funding is clustered in a handful of provinces and institutions, favouring biomedical research and disfavouring behavioural interventions, adaptation, and institutional response. Preference for biomedical research may be due to the detriment of clinical research.

## 1. Introduction

Government-sponsored medical research funding is always scarce, but recent policy decisions at the federal level have further constrained academic research budgets [1] such that funding priority setting has become a prime task for policy makers and institution administrators alike.

At the same time, more important challenges to population health are entering the public consciousness, and with them calls for deeper and broader investigations. One such challenge is autism spectrum disorder (ASD), whose prevalence in the Canadian population is largely unknown, but whose prominence in the national media and discourse is high, as is its perceived social burden. No other neurodevelopmental disorder has seen such a recent explosion in fundraising and lobbying activities [2]. In the USA, from 1995 to 2001, research funding for ASD quintupled from $US 11 million to $US 56 million [3]. The public demand, as least as seen through the eyes of parents with ASD children, appears to be for immediate breakthroughs in treatment and in utero detection [4, 5].

While it is important to understand the underlying biological basis of ASD, it is equally as important to understand the nonbiological risk factors for the disease, as well as opportunities for social or behavioural interventions, and economic factors useful for modelling the burden of disease. In other words, in an era of compressed research funding, attention must be paid to the domains of research within a given subject area that funding decisions tend not to reward. This is particularly relevant for funding decisions made by the Canadian Institutes of Health Research (CIHR), which is Canada's principle funding body for human health-related research.

Thus, with this study we examined the distribution and temporal trends in CIHR's funding grants for topics in ASD, for the purpose of refocusing the public debate on research priorities. We feel that this approach can be applied to other areas of health research that rely upon a small number of principle funding sources and is useful for achieving high level perspective on the extent to which funding decisions reflect national research values.

## 2. Methods

We conducted a search of CIHR's public funding database, which includes bilingual (English and French) information on successful grant applications, using the following keywords and their French equivalents: autism, autistic, ASD, Asperger syndrome, pervasive developmental disorder, PDD-NOS, Rett's syndrome, and Childhood Disintegrative Disorder. The initial search was conducted on Nov 28th, 2012, and then updated on June 2nd, 2013.

First, titles and abstracts were reviewed by a single investigator for probable relevance to ASD. In the event of uncertainty, the abstract was reviewed by a second investigator who then decided whether the project should be included in our set of studies to be analyzed. Grants that focused on multiple neurological disorders were disqualified. Grants were included if their abstracts indicated a dedicated focus on ASD or its effects and implications.

Demographic, geographical, thematic, and financial information were then extracted from the resulting grants. The collected variables are summarized in Table 1. Note that responses to “Theme of research” are coded in the funding database, having been preselected by each grant writer at the time of their project's submission to CIHR.

The distributive and temporal trends in funding were explored using descriptive statistics.

A sensitivity analysis was also conducted to determine if inclusion or exclusion of the grants reviewed by a second investigator would alter the results appreciably.

## 3. Results

Of 329 projects resulting from our keyword search, 252 were deemed relevant based upon their titles and abstract content, with 62 rejected due to lack of relevance and 15 rejected since there was no abstract to review. Of the 252 relevant grants, further 62 were excluded by the second reviewer due to lack of perceived relevance or due to lack of sufficient specific focus on ASD. In our sensitivity analysis, reinclusion of the latter 62 grants rejected by the second reviewer resulted in no appreciable change to our results, based upon a <5% change in total grant dollars.

Of all the years analyzed, the highest number of ASD-related grants (28) was funded in 2009, while 2003 saw the peak in the total dollar investment in ASD funding ($6.9 million).

A plurality of funding was allocated to biomedical research (see Table 2). Also, Ontario, Quebec, and British Columbia received 77% of all ASD-related grants (see Table 3). The lion's share of dollars, unsurprisingly, was granted by two CIHR Institutes: Neurosciences, Mental Health and Addiction (52%) and Human Development, Child and Youth Health (30%). The majority of grants were given for capacity building and knowledge creation purposes (see Table 4).

The top 3 institutions receiving the most money for ASD research were all in Ontario. Together, they received just over $19 million in ASD-related funding, which is almost 40% of the total dollars allocated nationally. Three institutions also received the most number of grants, accounting for 46% of all ASD-related grants. A few non-Canadian institutions, located in the USA and UK, were recipients of 6.9% of the grants.

In terms of temporal trends, both the number of grants and total funding dollars saw no obvious trend over the time period considered; however, both saw a steady yearly decrease from 2010 (27 grants totalling over $4 million) to 2013 (5 grants totalling under $1 million).

## 4. Discussion

According to our results, the lion's share of CIHR ASD funding is granted to researchers in Ontario and Quebec and to a handful of key institutions in those provinces. This is in line with the overall CIHR funding pattern, which dramatically benefits institutions in Ontario. A plurality of the ASD-related funding, in terms of both dollars (46%) and number of grants (43%), is earmarked for biomedical research, which is characterized by basic, “wet laboratory” studies, often dependent upon expensive equipment. And while overall ASD research funding has decreased over the past 3 years, most divestment has come from the areas of clinical, health systems, and social/cultural/population health research.

These results are somewhat aligned to similar findings in the USA [2], where funding data from the National Institutes of Health revealed that American ASD funding also favours basic medical research (65% of funded projects), though their system shows a steady growth in overall ASD funding, increasing at about 15% per year, while Canadian funding is in decline.

Our methodology suffers from a reliance on titles, abstracts, and researcher-chosen keywords to determine projects' applicability to ASD research. As well, we have no information on the number or nature of submitted or unfunded grant applications, raising the possibility that funding trends might actually correlate well with trends in actual researcher interest, but not in funder interest.

Most problematic is our reliance on a single source of research funding, specifically CIHR. However, in Canada the only major funders of ASD research outside of CIHR are a collection of autism-specific foundations, such as Autism Speaks Canada, the Spectrum Intervention Group, L'Amitient, the Canadian National Autism Foundation, and some international foundations with interest in Canada, such as the Geneva Centre for Autism. In 2012, these groups provided a combined total of just over $500,000 in research funding [6], which is significantly smaller than that provided by CIHR. The Ontario Brain Institute, as well, embraces a multidisciplinary funding approach to autism, though we could not identify the dollar amount offered by that funder.

In addition, for the last few years, CIHR has been responsible for disbursing all federal health-related research funds, whereas historically the Social Sciences and Humanities Research Council (SSHRC) might have funded some social/cultural projects related to ASD. A quick search of 1998–2013 SSHRC competition results, using the keyword “autism” without a deeper search for relevance, reveals about 100 funded grants totalling approximately $2 million, which is very small compared to CIHR funding over the same time period [7]. A similar search of the NSERC grants database found just over $3 million in monies associated ASD, which includes funds earmarked for both research and student support [8], though earlier records may not be accessible through NSERC's online portal.

While it would have been ideal to have included NSERC and SSHRC funding data in our general analyses, our initial intent was to specifically examine the extent to which CIHR ASD funding was aligned with societal priorities, with an understanding that CIHR's granting activities would be most reflective of the Canadian government's policies on this manner. Our cursory search of NSERC and SSHRC ASD-related activities indicated that those agencies' contributions were of insufficient size and scope to have altered our findings or conclusions.

It is worth pointing out that while our results suggest an overall national trend in focusing on the strict biomedical dimensions of ASD, there exist pockets of researchers who strive to more comprehensively approach disorders of national interest. A notable example is the Ontario Brain Institute, which embraces a multidisciplinary approach to disorders of the nervous system with an explicit focus on prevention, detection, and treatment, an approach that necessarily includes nonbench related science as well as basic biomedical research. Thus, our findings should not suggest that researchers in Canada are unaware of the need for a diverse and multidisciplinary approach to addressing a given health issue, but rather that funding priorities may be constraining the extent to which a diverse set of research tools is indeed being permitted to enter the fray.

Moreover, the importance of our results lies not in their description of funding patterns relating specifically to ASD, but rather in the implications for the extent to which these patterns reflect values underlying research priorities. Our finding that an increase in funding of what we call biomedical research, which seeks to explore the biological causes and treatments of the disease, coincides with a decrease in funding of projects seeking to address clinical (i.e., treatment oriented) or social/cultural/population aspects of the disease, which include studies on comorbidities, environmental associations, economic impacts, and social adaptation approaches. This is despite both literatures' stressing of the value of interventions to relive the stress of caregivers to ASD children [9] and the announcement of CIHR's doubling of its clinical research budget in 2012 [10]. While researchers and clinicians dealing with ASD typically do not view the disease through the dualistic lens that we have proposed, that of biomedical versus nonbiomedical approaches, it is our contention that the funding administrative dynamic artificially imposes such a false choice, necessitating the preference of one approach over the others. The underlying question is whether this funding preference is expressive of society's true priorities or is either an unintended consequence of the funding decision-making process or indeed a reflection of an unstated policy. This study, then, represents but one approach to explore this necessary alignment of research expenditure with societal priorities.

A similar approach was undertaken by Leroy et al. [11], who examined National Institutes of Health (NIH) and Gates Foundation granting data to determine the extent to which research funding aimed at reducing global child mortality was earmarked for technological improvement. They found that 97% of grants were for developing new technologies and expressed concern that insufficient research attention was thus being directed toward increased efficacy of treatment and delivery and use of technology. This discrepancy can be interpreted as a misalignment of funding priorities with both researcher values and the actual needs of the sector.

Scholarly research into the so-called “research values” is relatively scarce in the peer-reviewed biomedical literature. However, management and communication sciences have long acknowledged the extent to which social, political, and cultural agendas are necessarily reflected in money management strategies, including national research priorities. Despite this, there is conspicuous lack of agreement on what values actually are and the degree to which social values can affect individual decision making within the research milieu [12].

Accepting that Canadian values, expressed through research funding, are in some ways decided upon by Parliamentarians, researchers in 2007 measured Members of Parliament attitudes toward health research funding [13]. They found that while 84% of 308 federal Members of Parliament knew of CIHR, 32% knew nothing about its role; 66% believed that the business sector should be the primary source of health research funding; and 57% most frequently defined health research as study into cures or treatments of disease, rather than a disease's determinants, accommodations, role in societal interplay, sociolegal interactions, and so forth. Our results reflect a preference for a biological, cure-based research agenda and are thus seemingly in line with politicians' expectations, but there appears to be little opportunity or incentive for the private sector to fund the remaining types of research, as seemingly would be preferred by Parliamentarians. Seeking a cure is understandably society's priority for most illnesses. But diseases with strong lifestyle, psychological, social, and accommodative considerations, like ASD and other neurological disorders, distinct from dissimilar diseases, may have funding priorities beyond finding a cure or a preventative measure.

Perhaps an approach, like that pioneered by Sivananthan and Chambers [14], in which health research priority setting was systematically measured amongst researchers, caregivers, and policymakers, is warranted for a larger Canadian health research agenda. Similarly, Fleurence and Torgerson [15] point out that setting priorities for research should be conducted to make the most efficient use of scarce resources and that the uptake by policymakers of methods for identifying such priorities has been slow. In 2010, the World Health Organization had similarly conducted a process for identifying research priorities best aligned with its membership's values [16]. In the Canadian case, our national dialogue must go beyond prioritizing topics and delve into the intent of the research at hand, to determine the relative valuation of biomedical, clinical, health systems, and social/cultural/population health approaches for the long term wherewithal of our population.

To better address the issue, further investigations are warranted, preferably including funding sources beyond CIHR, embracing topics beyond ASD, and, critically, considering the nature of unfunded projects, as well, in order to determine whether patterns of funded research only reflect decision-makers' priorities, or whether the topics sought by the researchers themselves are also in line with these patterns. As health research funding dwindles, each nation is well-advised to consider the values that must determine the facets of individual health problems that are most important to its citizenry, and therefore most deserving of taxpayer money.

## Figures and Tables

**Table 1:** Variables extracted from CIHR funded grants.

Variable name	Description
Amount	Total amount of funds released by CIHR

Year	Fiscal year in which grant was awarded

Grant type	(i) Capacity building; (ii) knowledge creation; (iii) knowledge translation

Theme of research	(i) Biomedical; (ii) clinical; (iii) health systems/services; (iv) social/cultural/environmental/population health

Province	Province in which administering institution is located

CIHR Institute	CIHR institute from which funds originated: (i) Neurosciences, Mental Health and Addiction; (ii) Human Development, Child and Youth Health; (iii) Genetics; (iv) Health Services and Policy Research; (v) Aging; (vi) Population and Public Health

**Table 2:** Distribution by research theme of all grants and all ASD-related grants awarded by CIHR from 1991 to 2013.

Research theme	Percentage of number of ASD-related grants funded	Percentage of dollar value of ASD-related grants given	Percentage of dollar value of all CIHR grants given
Biomedical	43%	46%	53%
Clinical	27%	41%	13%
Not applicable or not specified	12%	3%	21%
Health systems/services	10%	7%	5%
Social/cultural/environmental/population health	8%	3%	8%

**Table 3:** Distribution by region of all grants and all ASD-related grants awarded by CIHR from 1991 to 2013.

Province	Percentage of number of ASD grants funded	Percentage of dollar value of ASD grants given	Percentage of dollar value of all CIHR grants given
Ontario	48%	60%	41%
Quebec	18%	18%	29%
British Columbia	11%	3%	12%
Alberta	7%	12%	10%
Nova Scotia	4%	2%	3%
The rest of the country and out of country	12% (no single province was >2%)	5% (no single province was >1%)	5%

**Table 4:** Distribution by type of CIHR ASD grants from 1991 to 2013.

Type of grant	Percentage of number of grants funded	Percentage of dollar value of grants given
Capacity building	44%	14%
Knowledge creation	31%	53%
Not classified	19%	31%
Knowledge translation	6%	2%
